# Assessing ecosystem vulnerability under severe uncertainty of global climate change

**DOI:** 10.1038/s41598-023-31597-6

**Published:** 2023-04-12

**Authors:** Tetsuro Yoshikawa, Dai Koide, Hiroyuki Yokomizo, Ji Yoon Kim, Taku Kadoya

**Affiliations:** 1 National Institute for Environmental Studies, 16-2 Onogawa, Tsukuba, Ibaraki 305-8506 Japan; 2 Graduate School of Science, Osaka Metropolitan University, 3-3-138 Sugimoto, Sumiyoshi-ku, Osaka, 558-8585 Japan; 3 Department of Biological Science, Kunsan National University, 558 Daehak-ro, Gunsan-si, Jeolabuk-do 54150 Republic of Korea

**Keywords:** Conservation biology, Climate-change ecology

## Abstract

Assessing the vulnerability and adaptive capacity of species, communities, and ecosystems is essential for successful conservation. Climate change, however, induces extreme uncertainty in various pathways of assessments, which hampers robust decision-making for conservation. Here, we developed a framework that allows us to quantify the level of acceptable uncertainty as a metric of ecosystem robustness, considering the uncertainty due to climate change. Under the framework, utilizing a key concept from info-gap decision theory, vulnerability is measured as the inverse of maximum acceptable uncertainty to fulfill the minimum required goal for conservation. We applied the framework to 42 natural forest ecosystems and assessed their acceptable uncertainties in terms of maintenance of species richness and forest functional type. Based on best-guess estimate of future temperature in various GCM models and RCP scenarios, and assuming that tree species survival is primarily determined by mean annual temperature, we performed simulations with increasing deviation from the best-guess temperature. Our simulations indicated that the acceptable uncertainty varied greatly among the forest plots, presumably reflecting the distribution of ecological traits and niches among species within the communities. Our framework provides acceptable uncertainty as an operational metric of ecosystem robustness under uncertainty, while incorporating both system properties and socioeconomic conditions. We argue that our framework can enhance social consensus building and decision-making in the face of the extreme uncertainty induced by global climate change.

## Introduction

**Figure 1:**
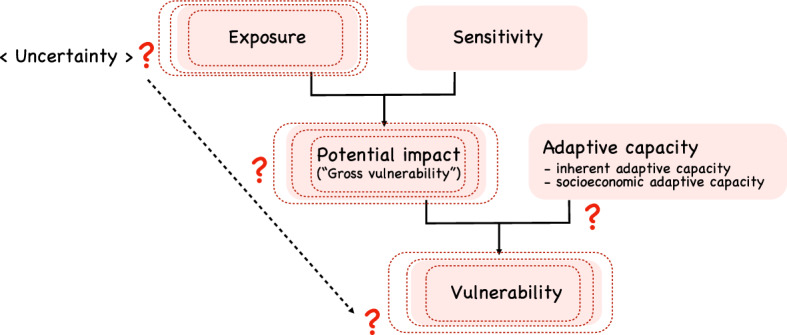
Standard framework of the exposure–sensitivity–vulnerability concept^4–6^. The potential impact (or “gross vulnerability”) of a system is determined by exposure to changing environments as well as by sensitivity of the system itself. The vulnerability is defined as potential impacts alleviated by the adaptive capacity of the system. Uncertainty is ubiquitous in each of the elements in this framework.

The progression of global climate change is the greatest threat to biodiversity and ecosystem services in both terrestrial and marine ecosystems^1^. Exposure to rapid changes in climate is causing species loss, community shifts, and community collapse^2–4^. Thus, for the successful conservation of species, communities, and ecosystems, we need ways to rapidly assess their vulnerability under future climatic conditions^5,6^. In sustainability research, vulnerability is defined as the degree of harm that a system experiences with exposure to an environmental change^7,8^. Vulnerability is usually conceptualized as the outcome of the combination of exposure (e.g., climate changes experienced by a system) and sensitivity (the degree to which a system is affected by exposure)^9^ (Fig. 1). However, vulnerability can be mitigated by adaptive capacity, a biological system’s inherent or external ability to cope with change due to exposure^7,8^ (Fig. 1). Assessing an ecosystem’s exposure, sensitivity, and adaptive capacity is essential for stakeholders and decision-makers when quantifying its anticipated vulnerability and prioritizing conservation targets and management actions to mitigate the vulnerability and conserve the ecosystem^7,10^.

Uncertainty is inevitable in the various pathways of vulnerability assessment of species, communities, and ecosystems^11^ (Fig. 1), especially as global environmental changes have been magnified. While the projection of future climate is an essential basis for such assessments, it often has propagated uncertainty, particularly at local spatial scales and at large temporal scales^12^. Projected spatial and temporal patterns of climate also vary greatly among general circulation models (GCMs) and future anthropogenic carbon emission scenarios. Although the 5th and later IPCC reports incorporated multiple global carbon emission scenarios (i.e., representative concentration pathways; RCPs), uncertainty among and within the scenarios remains large. Therefore, incorporating this uncertainty in models of climatic exposure is a critical challenge for robust assessment of ecosystem vulnerability and planning of management options^11,13–15^.

Various approaches have been used to consider the uncertainty of climate projection when assessing the future status of conservation targets. The use of several different climate models or scenarios is a basic approach, but the output is often difficult to use for decision-making when sufficient knowledge of the relative likelihood of each model or scenario is lacking^16^. Ensemble forecasting and probabilistic assessment^17,18^ can integrate outputs of multiple models while considering their relative likelihoods, and thus provide a better estimate of future climate or target responses. Even in these approaches, however, failure in the prediction at the focal spatial and temporal scales can mislead decision-makers to wrongly prioritize conservation targets and use unsuitable management options for them^19^. Thus, either of these approaches forces managers to plan highly flexible adaptive strategies, which are often difficult to implement^13,19^. Therefore, to cope with the uncertainty relating to climate projections, we need new ways to assess vulnerability^11^.

Info-gap decision theory (IGDT) is a promising approach to robust decision-making under extreme uncertainty of key conditions^20–22^. Instead of seeking a decision that is optimal under the best estimate of a key condition, the aim of IGDT is that a decision will maximize the likelihood of attaining a certain acceptable goal under uncertainty of the key condition^21^. The IGDT approach has been adopted in a wide range of fields where consideration of uncertainty is a key issue, including species conservation^19,23^, forest management^24^, and other resource management. Although the primary focus of the IGDT is on decision-making by comparing multiple strategies, the approach for measuring robustness applied by IGDT would also be valuable to learn the degree of acceptable uncertainty of a focal scenario under a given minimum requirement.

**Figure 2:**
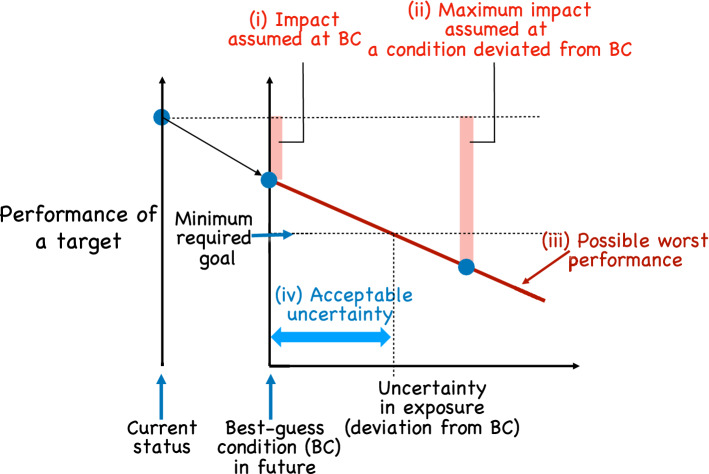
The proposed framework for vulnerability assessment under uncertainty in climate change predictions. In this figure, performance of a target system (e.g., ecosystem services or variables related to them) is expected to decrease from the current status under the future best-guess condition (BC). This decrease can be seen as an impact under BC (i). When a condition deviates from BC, and by focusing on the maximum impact within the deviation (degree of uncertainty) (ii), the possible worst performance decreases more (iii). When a minimum required goal is set, acceptable uncertainty for the minimum required goal can be determined (iv), which is a metric for the robustness (i.e., anti-vulnerability) considering uncertainty in exposure.

Here we propose a vulnerability assessment of ecosystems based on the IGDT framework (Fig. 2). In the framework, the state of the system (e.g., species richness) is first assessed to quantify the impact under the best estimate of a key condition (e.g., best probable climate change projection; (i) in Fig. 2). Next, the worst change in the state is evaluated by increasing the deviation from the best-guess condition (e.g., degree of uncertainty in the projection of temperature) to quantify the maximum impact within the uncertainty range ((ii) in Fig. 2). Through this procedure, the framework quantifies the relationship between degree of deviation from the best-guess condition and possible worst performance within the deviation range ((iii) in Fig. 2). This framework thereby enables us to quantify the maximum acceptable deviation from the best-guess condition attaining a minimum required goal in a comparable manner ((iv) in Fig. 2). The system with the smaller acceptable uncertainty is considered more vulnerable. Hence we propose the inverse of acceptable uncertainty as a metric of vulnerability.

As a case study for the proposed framework, we assessed the acceptable uncertainty of natural forests of various functional types against future climate change in Japan. Natural forest communities provide many ecosystem services, including carbon stock, nutrition cycling, mitigation of natural disasters, and maintaining biodiversity. Among the major forest ecosystem properties, species richness (degree of biodiversity) and forest type (dominant tree functional group, which characterizes physiognomy of the forest) are strongly associated with those essential ecosystem services^25^. Future climate change is expected to bring about species declines and forest type shifts in the majority of forest communities. Furthermore, since local climate projection in the Japanese archipelago is challenging owing to its complex topography, the assessment of ecosystem vulnerability under uncertainty has critical importance for forest ecosystem management and climate change adaptation. While specifically focusing on species richness and maintenance of forest type as indicators of forest state, we evaluated the acceptable uncertainty of 42 natural forests with regard to temperature deviation from the best future prediction based on GCM simulations.

Moreover, to demonstrate the usefulness of the approach in evaluating the effectiveness of management, we evaluated whether an ecosystem management practice can improve the acceptable uncertainty of the focal forests. Namely, we assessed the effect of conserving the juvenile tree layer of a forest to be a species source for the next generation of the forest community, which is an essential ecological process governing the future status of forest ecosystems^4^.

## Materials and methods

### Outline of the simulation

We assume that each tree species has its thermal niche, represented by mean annual temperature (MAT), and that a tree species survival is determined by the relationship between the species’ thermal niche range and MAT at a location. Though association of tree mortality with climate factors is complicated, being variable among regions or ecological contexts, increase in temperature is a principal determinant of tree mortality, being related to risk of drought. Since increase in temperature in various growing seasons can results in drought risks in tree species^26,27^, we adopted mean annual temperature as proxy of tree mortality risk, instead of maximum annual temperature. Thus, once the MAT of a forest plot falls outside the species’ thermal niche range, the species is lost from the forest community.

Using this assumption, we simulated tree species loss at the best-guess condition based on future climate projection and at deviated conditions. The degree of species loss at the best-guess condition of future climate is considered as vulnerability in ordinary studies. We then simulated how species loss would increase with increasing uncertainty of MAT to identify the worst case scenario. We also traced the shift of functional types in the forests. The functional type of a forest is defined on the basis of its most dominant type of four functional types (explained later). In our simulation, entries of new canopy species into a plot are not considered, because establishment of new canopy species in the adult tree layer is difficult in the short period supposed in this study (about 30 years). We also ignored increments of basal area of individual trees from the current to future status, since they are too small within a 30-year interval.

We set the first series of goals of forest conservation as maintaining 90%, 75%, and 50% of the initial number of canopy tree species. The second goal is to maintain the initial forest type of each plot, such that the most dominant functional type (based on total basal area [TBA] of each type) is not altered by future climate change. We evaluated the acceptable deviation *k* for each plot, and thus considered it as an inverse vulnerability measure. In other words, a larger *k* value indicates less vulnerability under climate uncertainty.

### Application of the framework to Japanese forest plots

**Figure 3:**
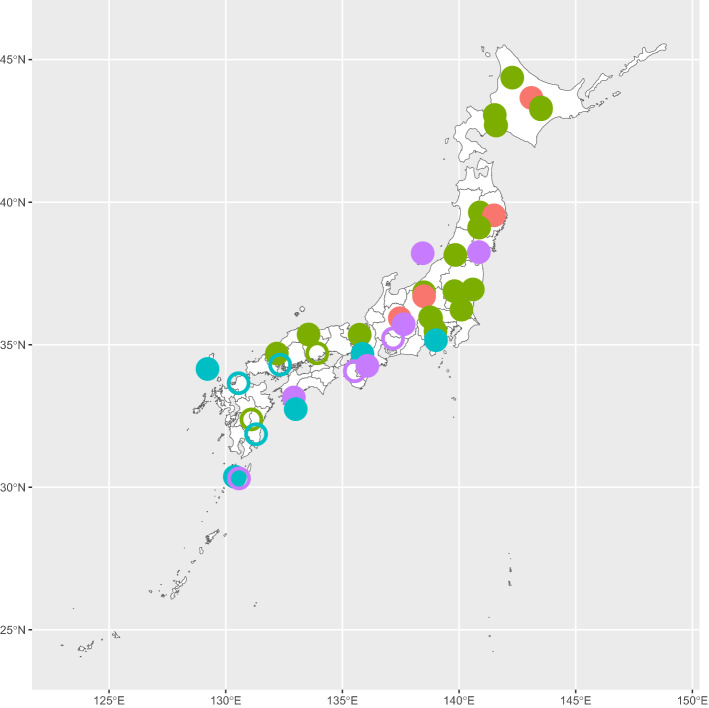
Distribution of the focal 42 forest plots across the Japanese archipelago. Point color and shape correspond to forest type and forest age, respectively. Red, boreal coniferous forest; green, deciduous broadleaved forest; blue, temperate coniferous forest; purple, evergreen broadleaved forest. Filled circle, old-growth forest; empty circle, secondary forest.

We assessed the vulnerability of 42 Japanese forest communities of various forest types (Fig. 3). Each of the forest communities has been measured in a 1-ha plot as part of the Monitoring Site 1000 project led by Japan’s Ministry of Environment. The plots include both old-growth forests that had not experienced large disturbance in 150 years and secondary forests that had been established within the last 150 years. In each plot, all the stems with ≥ 15 cm girth at breast height were measured at intervals of one to several years beginning in 2004^28,29^. In each forest, we selected the data measured in a single year between 2011 and 2015 as the current status of the forest. Because non-canopy species make little contribution to the TBA, we focused only on canopy tree species (i.e., those with the potential to reach 10 m in height) and extracted data for those adult individuals with ≥ 10 cm diameter at breast height (DBH), for a total of 176 canopy tree species in all the plots.

We determined the forest type of each plot from the composition of tree functional types. Tree species in Japan are usually classified into four functional types: boreal conifer, cool-temperate deciduous broadleaved tree, temperate conifer, and warm-temperate evergreen broadleaved tree^29^. These functional types generally characterize typical forest types or forest biomes in the Japanese archipelago. We calculated the TBA of each functional type in a plot, and classified the functional type with the largest TBA as the forest type. Numbers of the plot characterized by boreal conifer, deciduous broadleaved tree, temperate conifer, and evergreen broadleaved tree are 4, 21, 9, and 8, respectively. The 42 plots included 32 old-growth forests, including 4 boreal conifer, 17 deciduous broadleaved, 6 temperate conifer, and 5 evergreen broadleaved plots. Plants of other types (e.g. bamboos) are very rare and negligible in these plots.

### Thermal niche of tree species

The thermal niches of the 176 canopy tree species recorded in the 42 plots were estimated from their presence data in 6th and 7th National Vegetation Survey data (Biodiversity Center, Nature Conservation Bureau, Ministry of the Environment; http://www.biodic.go.jp/​trialSystem/EN/info/vg67.html). These national surveys recorded presence of plant species between 1999 and 2013 at 33,487 points established throughout the Japanese archipelago. All 176 tree species found in the forest plots were recorded in these surveys. From the nationwide 1-km^2^ MAT data for 1981–2000^30^, we calculated each species’ thermal niche as the range between the 95% quantiles of MAT at the sites where the species was recorded. For tree species with fewer than 50 recorded sites, the thermal niche was defined as the range between maximum and minimum MATs of the recorded sites. Some species are distributed out of the range of the national vegetation survey, and our estimated thermal niches may underestimate actual ones. Nevertheless, since Japan is an island country where species have long-isolated populations and our target plots are distributed in mainly central islands, these potential biases are minimized.

### Projection of future temperature

Projection of future temperature varies among the GCMs used as well as among RCPs, yielding large uncertainty. Using the projected climate data (1 km × 1 km resolution) of 2031–2050 provided by Ishizaki et al.^30^, we calculated the average of the values of all possible combinations of four GCMs (MIROC5, GFDL-CM3, HadGEM2-ES, and MRI-CGCM3) and the two RCP scenarios (RCP 2.6 and RCP 8.5) for each forest. We adopted this average value of temperature between 2013–2050 as the trend mean of MATs (hereafter TM_MAT_), which is the best-guess condition of 2031–2050 MATs of the plot.

### Species richness at TM_MAT_ in the future

First, we simulated species survival/loss in each forest at its TM_MAT_. We assumed that a tree species cannot inhabit a forest when the species’ thermal niche range does not overlap with the TM_MAT_ of the site. Under this condition, we traced species loss with increasing temperature deviation *k* from TM_MAT_. We increased *k* by a unit of 0.1 °C, then evaluated the worst species loss. That is, tree species whose thermal niche ranges do not overlap with the range between (TM_MAT_ − *k*) and (TM_MAT_ + *k*) are lost from the forest. As *k* increases, the number of surviving species is expected to decrease. Recall the possible worst performance line in our framework ((iii) in Fig. 2), and note that we eliminated the possibility of recruitment of new species.

We explored maximum *k* values (i.e., acceptable uncertainty; (iv) in Fig. 2) when 90%, 75%, and 50% of the original number of species at TM_MAT_ in the forest were maintained in each plot (i.e., minimum required goal in Fig. 2).

We also traced the shift of forest type when temperature reaches TM_MAT_ and when temperature deviation *k* increases. At each *k* value, forest types were classified on the basis of the surviving tree species. We assessed at which *k* value an initial forest type shifts to another type. This is a simplified scenario that ignores tree stem growth, but it is partly supported by monitoring data on Japanese forests that confirm that forest type rarely changes within 30 years only via natural stem growth (Yoshikawa et al., unpublished data).

### Forest management option and its influence on vulnerability

We considered the conservation and maintenance of the juvenile tree layer (5–10 cm DBH) as one feasible management action to mitigate forest vulnerability and evaluated its contribution to the acceptable uncertainty of the forests. In this scenario, we assume that all species present in the juvenile layer can recruit into the adult layer in a plot only when sufficient management actions are performed. In addition to the abovementioned simulation of adult trees (DBH > 10 cm), we performed the same simulation for the survival of species in the juvenile tree layer (DBH ≤ 10 cm). With proper management, we assume that the surviving juvenile trees, which include species absent in the adult tree layer, can grow up and thus can compensate for species loss in the adult tree layer. We explored whether this juvenile layer management can alleviate species loss and prevent forest type shift in the simulation.

### Ethical approval

Experimental research and field studies on plants (either cultivated or wild), including the collection of plant material, complies with relevant institutional, national, and international guidelines and legislation.

## Results

### Vulnerability assessment of species richness and forest type in the best-guess condition

**Figure 4:**
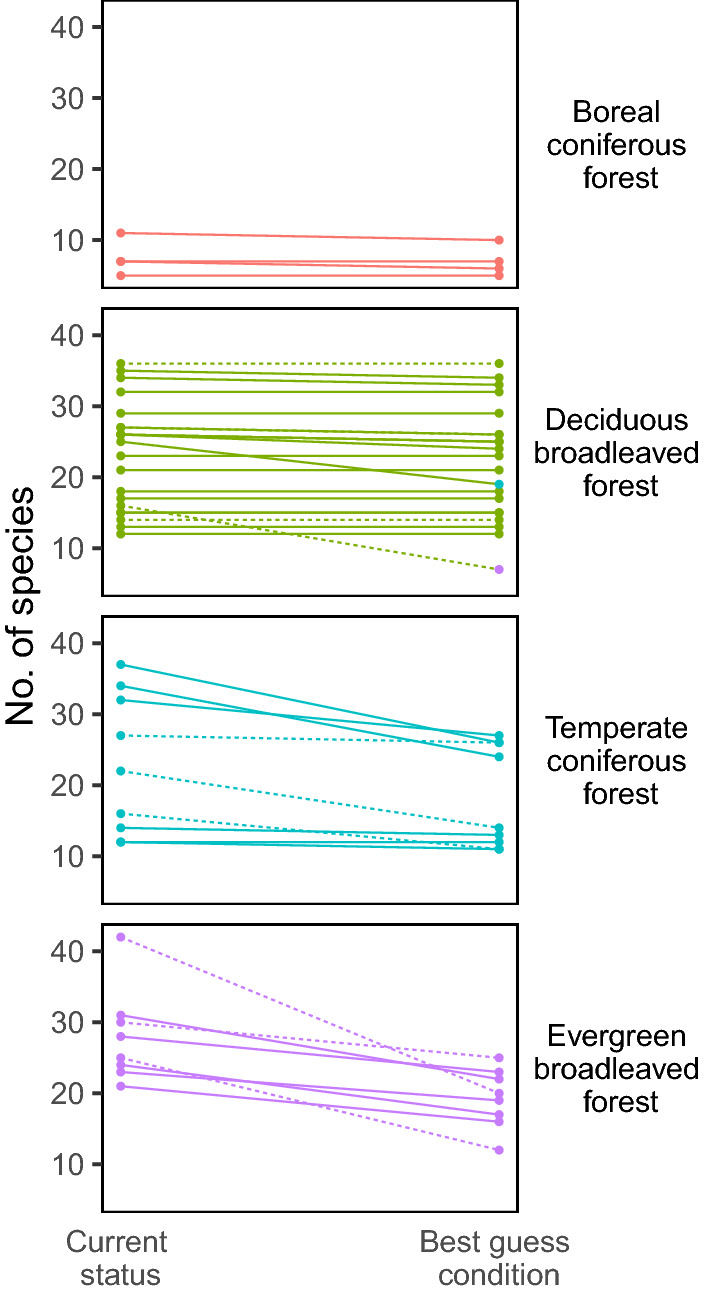
Species richness of the 42 forest plots under the current status (left) and under the projected trend mean of mean annual temperature (TM_MAT_) in 2031–2050 (right) in the plots of each forest type. Solid lines, old-growth forests; dashed lines, secondary forests. Point color indicates forest type (see Fig. 3). Two plots of deciduous broadleaved forests shifted functional types at TM_MAT_ (based on total basal area of surviving tree individuals), which is indicated by different point colors on the right side.

Initial species number (> 10 cm DBH) in each plot ranged from 6 to 48, differing among forest functional types (*p* < 0.001) but not between forest ages (*p* = 0.652) (two-way ANOVA; Fig. 4). The proportion of species predicted to be lost at TM_MAT_ of 2031–2050 was 0.13 ± 0.16 (mean ± SD), ranging from 0.00 to 0.56. This value differed significantly among forest functional types (*p* < 0.001) and between forest ages (*p* = 0.011). The proportion of predicted species lost was largest in evergreen broadleaved forest plots and smallest in boreal coniferous plots (*p* < 0.0001; pairwise *t* test with Bonferroni adjustment). It was larger in secondary forests than in old-growth forests. At the TM_MAT_ of 2031–2050, forest functional type changed in only two deciduous broadleaved plots: one converted to temperate coniferous forest and the other to evergreen broadleaved forest (Fig. 4). However, other types of plots did not experience a change in the dominant functional types at the TM_MAT_ of 2031–2050.

### Acceptable uncertainty of species richness and forest functional type in the 42 forests

**Figure 5:**
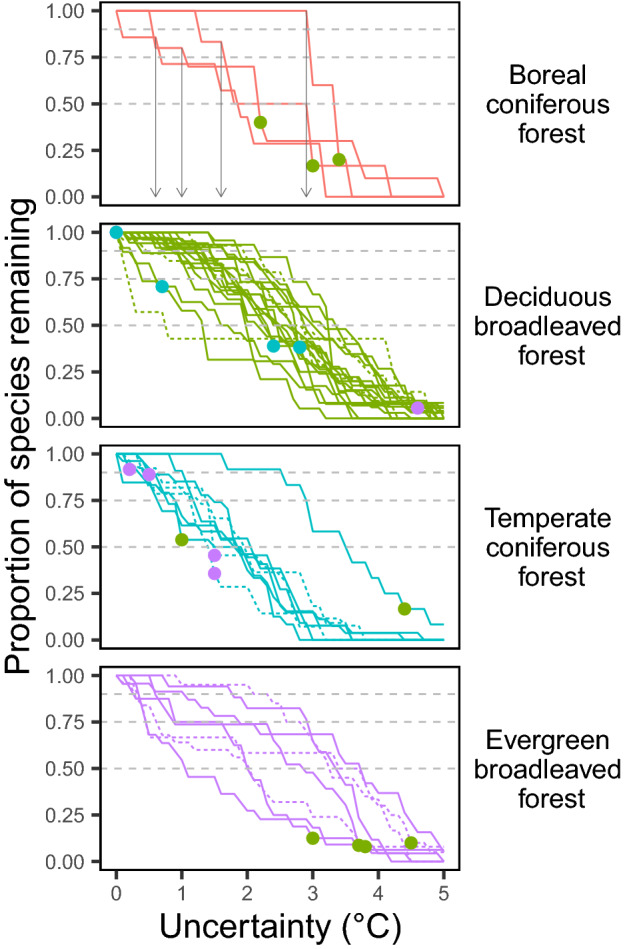
Species richness of the 42 forest plots at their worst-case scenarios with increasing uncertainty in TM_MAT_ (*k*). Dashed horizontal lines indicate 90%, 75%, and 50% of the initial species richness maintained at TM_MAT_. Points on the lines indicate change from the initial forest type, and the color indicates the shifted forest type (see Fig. 3). In the top panel showing the four boreal coniferous forest plots, arrows indicate maximum acceptable uncertainty (0.1 °C interval) for 75% species maintenance in each plot.

**Figure 6:**
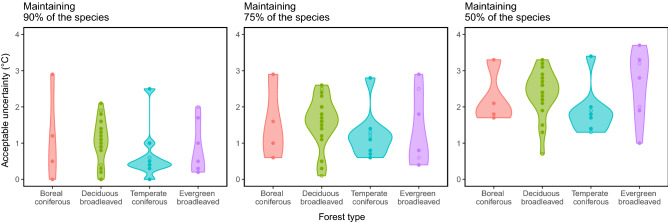
Acceptable uncertainty in TM_MAT_ for species richness maintenance for different forest types with different conservational goals: 90%, 75%, and 50% of species richness maintenance. Filled circles, old-growth forest; empty circles, secondary forest. Violin plots illustrate distributions of all the forest plots in the categories.

Robustness to climate uncertainty for species richness varied among the forest plots. As uncertainty around the TM_MAT_ increased, the species number was expected to decline in the worst case scenario (Fig. 5). These plots showed different declining patterns and different acceptable uncertainty to attain a fixed minimum goal of species richness (Figs. 5, 6); the acceptable uncertainty ranged from 0.0 to 2.9 °C (mean ± SD: 0.92 ± 0.72 °C) for 90% species maintenance, from 0.1 to 2.9 °C (1.45 ± 0.76 °C) for 75% species maintenance, and from 0.7 to 3.7 °C (2.30 ± 0.73 °C) for 50% species maintenance. However, these variations in the acceptable uncertainty for species richness (Fig. 5) were not explained by the forest functional types or forest ages of the plots (two-way ANOVA; *p* > 0.10).

Robustness of forest type change to climate uncertainty also varied among the forest plots. With increasing uncertainty around the TM_MAT_, forest type increasingly changed (Fig. 5). Before the uncertainty in TM_MAT_ reached 5.0 °C, about half of the plots experienced a change of forest type in their worst-case scenarios (19 plots: 3 boreal coniferous, 6 deciduous broadleaved, 6 temperate coniferous, and 4 evergreen broadleaved; Fig. 5). The probability of a plot experiencing forest type change did not differ among the initial forest types or between forest ages (χ^2^ test; *p* = 0.127 and *p* = 0.28, respectively). For these type-changing plots, acceptable uncertainty in TM_MAT_ was 2.27 ± 1.58 °C (range: 0.0–4.6 °C). When analyzing only these type-changing plots, we noted no significant difference in acceptable uncertainty among the forest types or between forest ages (two-way ANOVA; *p* > 0.15). Even when simulating under a single RCP scenario (RCP 2.6 or RCP 8.5), similar results were obtained (Supplementary Figures).

### Effects of juvenile tree conservation on acceptable uncertainty

**Figure 7:**
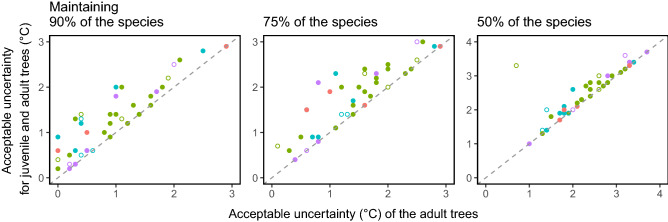
Effects of conservation of juvenile trees (5–10 cm DBH) on acceptable uncertainty in TM_MAT_ for species richness maintenance. The *x*-axis indicates acceptable uncertainty for communities of trees with > 10 cm DBH, and the *y*-axis indicates that for communities including both adult and juvenile trees. Point color and shape correspond to the forest types and ages (see Fig. 3).

When successional recruitment of all juvenile trees into the adult layer was assumed in the simulation, it increased the acceptable uncertainty for species richness maintenance (Fig. 7). The increment of acceptable uncertainty due to juvenile tree recruitment was 0.34 ± 0.33 °C for 90%, 0.33 ± 0.36 °C for 75%, and 0.18 ± 0.42 °C for 50% of species richness maintenance, which were all significantly larger than 0 (*p* < 0.01; *t* test). The increment of acceptable uncertainty did not differ among the forest types (*p* > 0.38 for all), but it did differ between forest ages (*p* = 0.04), with larger values in secondary forest plots. However, when a single outlier deciduous broadleaved plot was excluded, no difference was detected between the forest ages (*p* = 0.59).

**Figure 8:**
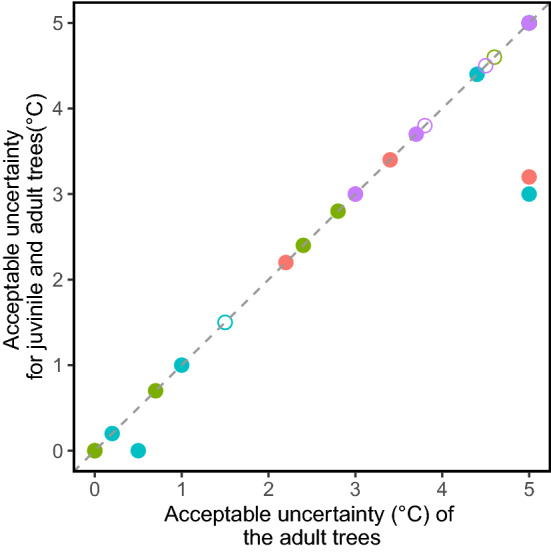
Effects of conservation of juvenile trees (5–10 cm DBH) on acceptable uncertainty in TM_MAT_ for forest type maintenance. The *x*-axis indicates acceptable uncertainty for communities of trees with > 10 cm DBH, and the *y*-axis indicates that for communities including both adult and juvenile trees. Point color and shape indicate functional type and forest age of the adult tree layer at the current stage (see Fig. 3).

However, simulated recruitment of all juvenile trees did not increase acceptable uncertainty for forest type maintenance. In fact, it reduced it in three plots, facilitating forest type conversion (Fig. 8).

## Discussion

By utilizing the key concept of the IGDT, we devised a framework to quantify the acceptable uncertainty as a metric of robustness of ecosystems, while considering the uncertainty due to climate change. We demonstrated the applicability of the proposed IGDT-derived framework using Japanese forests as a case study. The analysis showed that the forests have varying degrees of robustness to climate uncertainty, even within the same forest type or age class (Figs. 5, 6), and this variation overwhelms the differences in the proportion of surviving tree species in the best-guess condition at TM_MAT_ of 2031–2050 (Fig. 4). Thus, our approach can reveal vulnerabilities that are likely to be overlooked by a standard assessment, especially under the extreme uncertainty of climate change. The large difference in the acceptable uncertainty among the target Japanese forests may be due to the within-community distribution pattern of temperature niches of tree species or to functional diversity of traits related to these niches. Further, we speculate that low redundancy in temperature niches among community members would contribute to the robustness of species survival in the face of large climate uncertainty^31^. Although testing this expectation is beyond the scope of this study, future research should examine how acceptable uncertainty quantified by our approach is related to ecological trait and niche distributions among species in a community. Such research could help to clarify the relationships among ecological trait and niche distributions, vulnerability, and resilience of the community. We should also note that although we did not include the decision-making process explicitly in the present study, which is a primary focus of the IGDT, the decision-making process can be seamlessly incorporated into our framework whenever required such as decision-making on which forest to be managed in terms of overall forest state since we use the same framework with the IGDT in assessing the system’s behavior against uncertainty.

The juvenile tree layer of a forest community is a reservoir of its future generation and therefore directly affects the future forest composition^32^. Management of juvenile trees is a practical and feasible method to change the forest community trajectory and future forest status. However, these juvenile tree communities are susceptible to and exposed to browsing by large herbivores such as sika deer in Japan^33^. Our simple simulation on the effects of juvenile tree management demonstrated that successful recruitment of juvenile trees to the adult tree layer can increase acceptable uncertainty for the conservational goal of species richness maintenance (Fig. 6). Compositional differences between the juvenile and adult layers are likely to be a good indicator of a positive effect of the juvenile layer on acceptable uncertainty of the forest, because a juvenile tree layer can compensate for potential loss of species in the adult layer. On the other hand, success in recruitment of juvenile trees did not contribute to the maintenance of forest type, and it was shown to facilitate forest type change in some plots (Fig. 7). This seems reasonable, because the juvenile tree layer can include trees of different functional types than the dominant type in the current adult tree layer. Although forest type transition can certainly change and sometimes degrade ecosystem functions of a forest, the transition to an alternative vegetation type can be considered as a successful adaptation to environmental change when the maintenance of vegetation cover, for example, is the main concern^4^. Although acceptable uncertainty is defined for one performance, our approach can incorporate the multifaceted nature of forest adaptability and resilience by using multiple performance axes and minimum required goals in examining acceptable uncertainty of the focal ecosystem.

We should note, however, that our simulation for the case study on the Japanese forests is based on simplified assumptions in which tree species survival is determined only by a combination of local MAT and species thermal niche. In real ecosystems, tree growth and survival can be influenced by ranges of temperature, precipitation, and snowfall, as well as extreme values of these climatic variables and interspecific interactions. Maximum annual temperatures in combination with drought can be a relevant climate variable in forests in Europe and America. However, relationships between forest mortality and climate variables are not straightforward^26^; forest mortality is sometimes caused by high temperature in other seasons. In the consideration of these potential complexity, we adopted mean annual temperature as proxy of the occurrence of these extreme temperatures in various seasons, and as a main determinant of forest loss^26,27^. Interactions with other tree species can be another critical determinant for species survival/loss; species may persist outside its normal range due to facilitation among neighboring species^34^. Thus, these species interaction need to be considered for vulnerability assessment in future studies. Furthermore, phenotypic variability and plasticity of tree species can play an important role in adaptation to changing climates^35,36^. Considering these complex determinants of tree survival could yield different results regarding the vulnerability of communities. Nevertheless, our novel framework is valid for quantifying vulnerability related to uncertainty, even if more complex modeling approaches are performed, because it is general and can accommodate any types of models or systems.

Our framework quantifies the acceptable uncertainty as a metric of ecosystem robustness that considers the severe uncertainty in climate change. The acceptable uncertainty is determined by both the properties of the focal system (sensitivity) and social context (types of system performance and minimum required goal for the performance). In this respect, we would further argue that the inverse of acceptable uncertainty can be regarded as an integrated metric of vulnerability that considers not only uncertainty but also adaptive capacity. Specifically, the adaptive capacity that is inherent in the focal system is directly considered by the procedures in our analysis, in which the system behavior is thoroughly examined under a wide range of uncertainty. Furthermore, the adaptive capacity in the socioeconomic context is also considered in our framework as the minimum required goal of the performance. Socioeconomic adaptive capacity has critical importance for adaptation to climate change, since it has great influence on acceptable uncertainty. As conceptualized in Fig. 2, changing the goal greatly affects the acceptable uncertainty. In general, the socioeconomic condition that allows acceptance of a lower goal attains larger acceptable uncertainty and is robust to future climate change. Thus, our framework provides an operational metric incorporating the adaptive capacity, which facilitates consideration of the interaction between system properties and socioeconomic conditions. This framework should further enhance social consensus building and decision-making in the face of extreme uncertainty of conditions induced by global climate change.

## Supplementary Information

Supplementary Figures.

## Data Availability

The climate data used are published online in the webpage of the National Institute for Environmental Studies (https://www.nies.go.jp/doi/10.17595/20210501.001-e.html). Tree measurement data and tree distribution data are published online on the website of the Monitoring Site 1000 Project (https://www.biodic.go.jp/moni1000/) and that of the Biodiversity Center of Japan (https://www.biodic.go.jp/kiso/fnd_list_h.html/), respectively.
