# Supplementary material for: Assessing ecosystem vulnerability under severe uncertainty of global climate change
Source: Sci Rep. 2023 Apr 12;13:5932. doi: 10.1038/s41598-023-31597-6 (PMC10097691; doi:10.1038/s41598-023-31597-6)

Assessing ecosystem vulnerability under severe uncertainty of global climate change

Tetsuro Yoshikawa, Dai Koide, Hiroyuki Yokomizo, Ji Yoon Kim, Taku Kadoya

Supplementary Information

Figure S1.

Species richness of the 42 forest plots of each forest type under the current status and under the projected trend mean of mean annual temperature (TM_{MAT}) in 2031–2050 for RCP2.6 (left, a) and RCP8.5 (right, b). Solid lines, old-growth forests; dashed lines, secondary forests. Point color indicates forest type (see Fig. 3). Two plots of deciduous broadleaved forests shifted functional types at TM_{MAT} (based on total basal area of surviving tree individuals), which is indicated by different point colors on the right side.

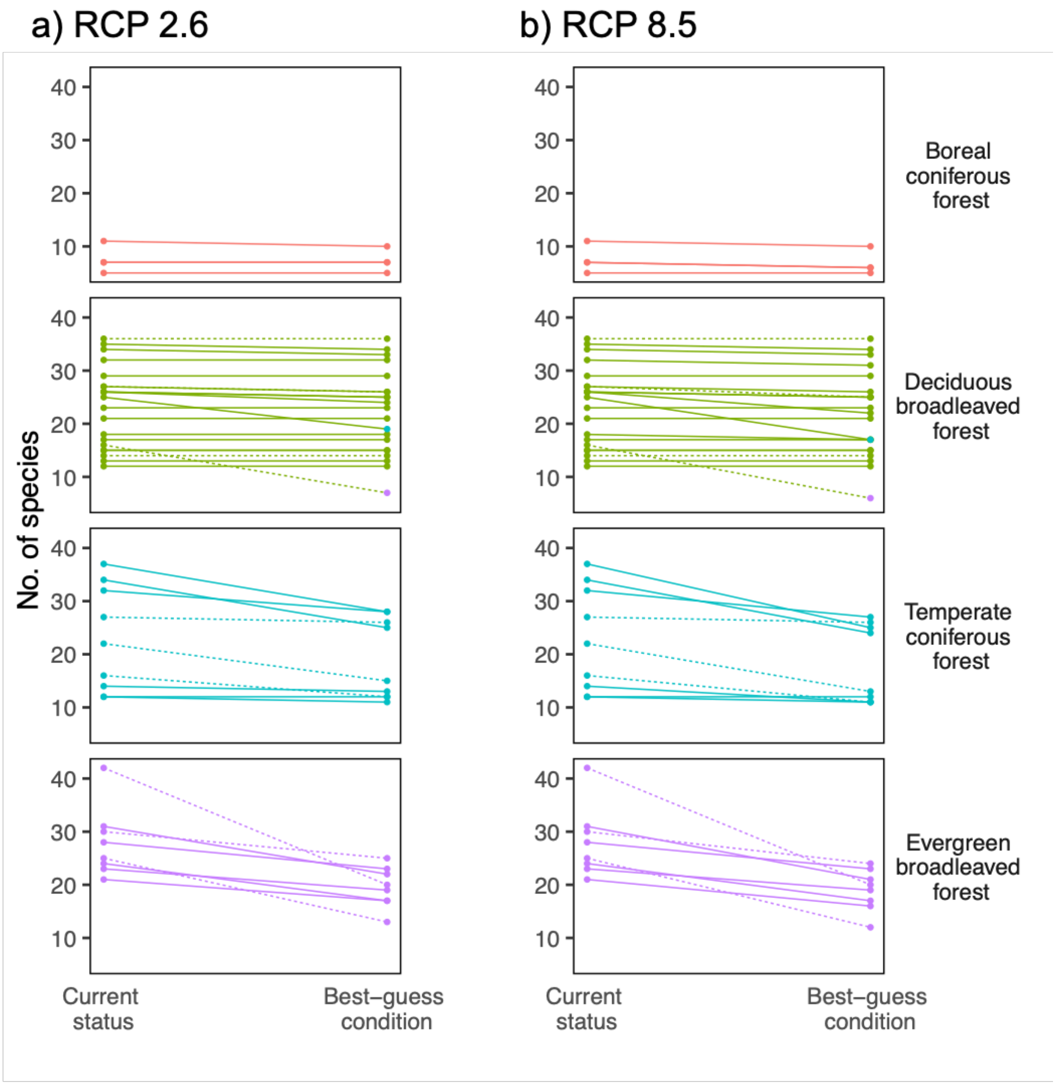

Figure S2.

Species richness of the 42 forest plots at their worst-case scenarios with increasing uncertainty in TM_{MAT} (k) for RCP2.6 (left, a) and RCP8.5 (right, b). Dashed horizontal lines indicate 90%, 75%, and 50% of the initial species richness maintained at TM_{MAT}. Points on the lines indicate change from the initial forest type, and the color indicates the shifted forest type (see Fig. 3).

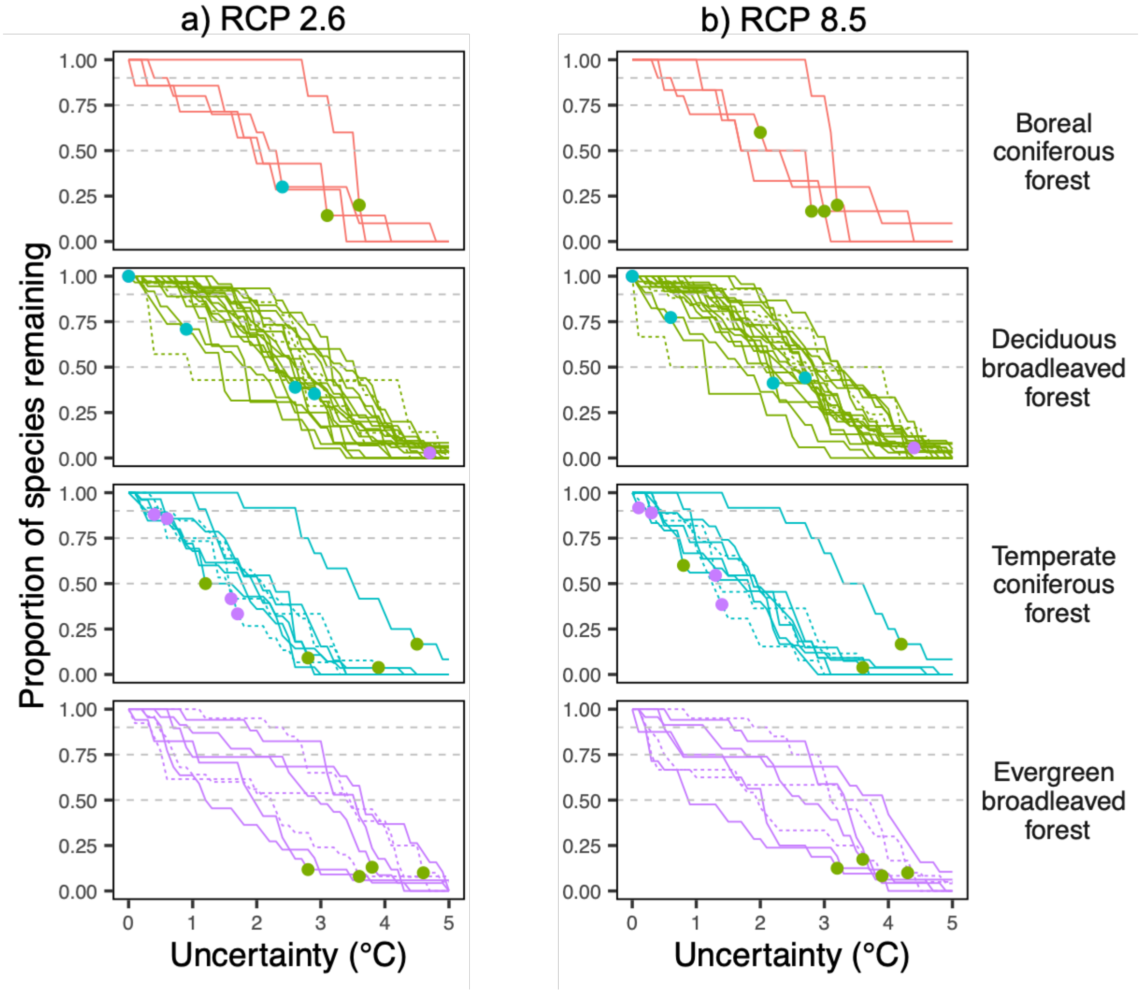

Figure S3.

Acceptable uncertainty in TM_{MAT} for species richness maintenance for different forest types with different conservational goals: 90%, 75%, and 50% of species richness maintenance. Upper graphs are results of RCP 2.6, and bottom RCP 8.5. Filled circles, old-growth forest; empty circles, secondary forest. Violin plots illustrate distributions of all the forest plots in the categories.

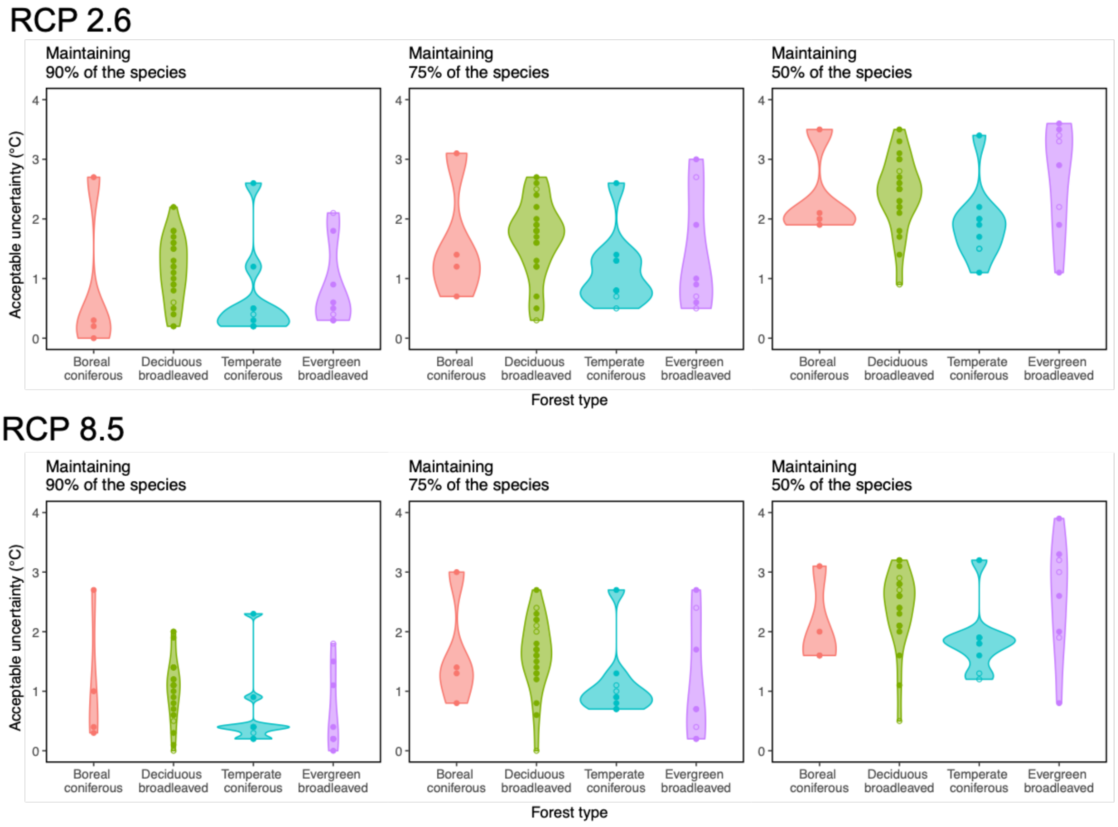

Supplement: Supplementary file 1 — Supplementary Figures. [file 41598_2023_31597_MOESM1_ESM.pdf]
